# Ectopic pregnancy in left ovary and contralateral uterine tube diagnosed one week apart in *In Vitro* Fertilization with donor eggs: Case report

**DOI:** 10.5935/1518-0557.20190030

**Published:** 2019

**Authors:** Vanessa Devens Trindade, Lauren Burmann, Dieny Viégas, Marta Ribeiro Hentschke, Ricardo Azambuja, Lilian Okada, Rafaella Gehm Petracco, Alvaro Petracco, Mariangela Badalotti, João da Rosa Michelon

**Affiliations:** 1 Fertilitat – Centro de Medicina Reprodutiva, Porto Alegre, Rio Grande do Sul, Brasil; 2 Pontifícia Universidade Católica do Rio Grande do Sul. Porto Alegre, Rio Grande do Sul, Brasil

**Keywords:** pregnancy, ectopic, fertilization *in vitro*, laparoscopy, infertility, case report

## Abstract

Bilateral ectopic pregnancy is a rare clinical condition with an estimated prevalence of 1/200 000 in spontaneous pregnancies. Studies have found that In Vitro Fertilization (IVF) is related to ectopic pregnancy independently, but the incidence of tubal disease in the donor egg recipient population is thought to be significantly lower than in the standard IVF population. We report the case of a patient participating in the egg-sharing program, who was diagnosed with ovarian ectopic pregnancy, treated with surgery. After one week, she was diagnosed with tubal ectopic pregnancy in the contralateral tube. The clinician should be aware that the treatment of one ectopic pregnancy does not preclude the occurrence of a second ectopic pregnancy in the same patient and should pay attention to the intra-operatory inspection of both side fallopian tubes in any ectopic pregnancy case. Routine ultrasound after ectopic pregnancy treatment may be reasonable, especially in high risk patients.

## INTRODUCTION

Ectopic pregnancy is an important cause of maternal morbidity and mortality, with an incidence oscillating between 1% - 2% of all reported pregnancies. Previous ectopic pregnancy, genital infection, pelvic inflammatory disease, tubal disease, abdominal surgery and smoking are directly associated with future ectopic pregnancies (Farquhar, 2005), while other risk factors such as assisted reproductive technology (ART) may be indirectly associated with this condition. Bilateral ectopic pregnancy is a rare condition, with an estimated prevalence of 1/200000 spontaneous pregnancies (Polat *et al.*, 2014). Tubal disease has been identified as one of the most significant risk factors for tubal pregnancy (Rosman *et al*., 2009). The incidence of tubal disease in the donor egg recipient population is thought to be significantly lower than in the standard IVF population, which would presumably lead to a lower rate of ectopic pregnancies in this group of patients. However this issue is controversial (Rosman *et al*., 2009). Herein we aim to describe a case of bilateral ectopic pregnancy diagnosed one week apart in a patient who performed *In Vitro* Fertilization (IVF) with donor eggs.

## CASE REPORT

A 43-year-old woman, Caucasian, living in the south of Brazil, sought an assisted reproduction center because of her desire to gestate. Faced with a history of endometriosis associated with decreased ovarian reserve due to her age, the patient underwent ART. She referred to have performed a laparoscopy due to infertility previously, when bilateral tubal permeability was identified. Initially, she began ovarian stimulation, but her cycle was canceled due to poor response. After two failed ovarian stimulations, she entered the "egg-sharing program". At that time, the egg-sharing program was the only allowed method of receiving egg donation in Brazil. Nowadays, a patient can spontaneously donate eggs. The selected donor was 34 years old and patient received nine mature oocytes from her. Four oocytes fertilized and two freshly fertilized embryos were transferred on cleavage state (D3), under ultrasonographic guidance. The endometrium thickness at the day of transference was 9.6 mm. As the standard laboratory protocol, embryos were loaded and transferred in 5 µL of culture medium (Global medium, Brussels/Belgium). The couple had no surplus embryos to freeze. The patient conceived and the Human chorionic gonadotrophin, Beta fraction (βhCG) level was 76.4 mIU/mL on the 12^th^ day after transfer.

**Figure 1:**
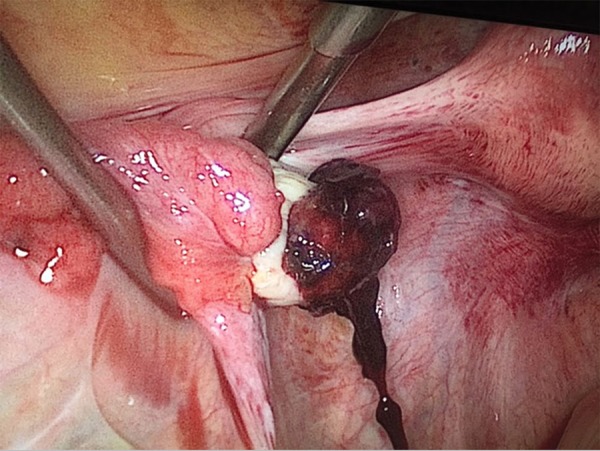
Left ovarian ectopic pregnancy 25 days after embryo transfer

**Figure 2:**
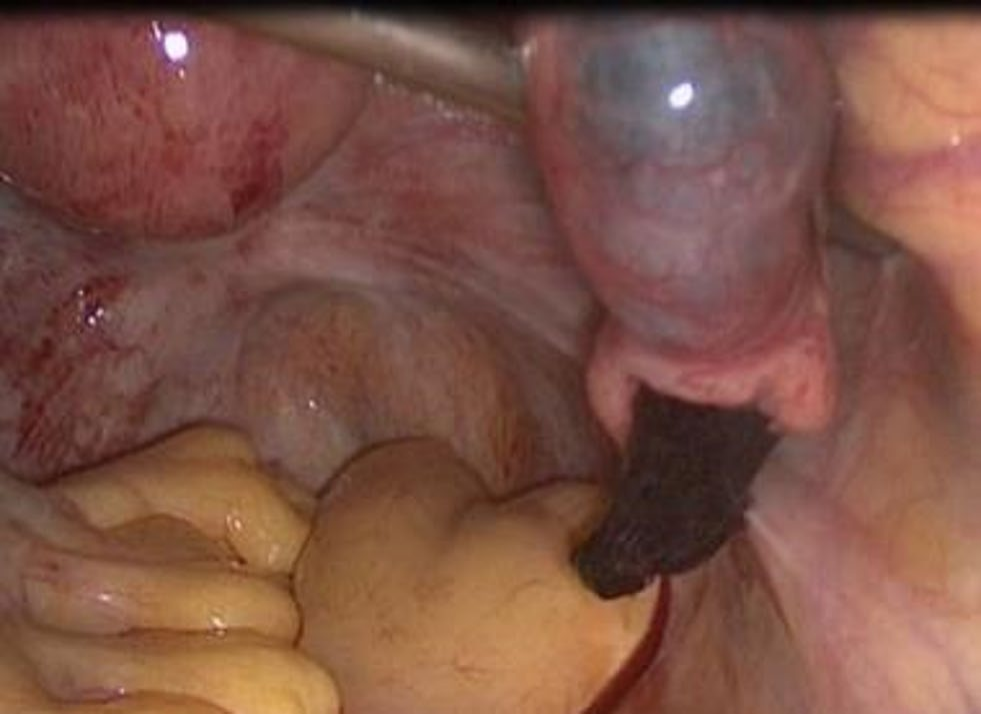
Right tubal ectopic pregnancy 33 days after embryo transfer

On the 25^th^ day after transfer, before performing ultrasound control, patient sought medical help with abdominal pain and vaginal bleeding, hemodynamically stable. A transvaginal pelvic ultrasound examination revealed an empty uterus without any adnexal masses and an important amount of fluid collection was presented in the pouch of Douglas. The medical team decided to perform a diagnostic laparoscopy. Surgery revealed hemoperitoneum of approximately 800 mL. There was an ovarian ectopic pregnancy on the left ovary (Figure 1), measuring about 20 millimeters in diameter. Active bleeding was identified and the surgeons performed excision of ectopic tissue with preservation of the affected ovary. The right ovary was of usual aspect and normal tubes were identified on both sides. Patient was discharged in good general condition on the same day. After 8 days, patient reported a new acute abdominal discomfort. A transvaginal ultrasound examination revealed an empty uterus again, but with a right side adnexal mass of 20 millimeters along with an important amount of free fluid in the pouch of Douglas. She was hemodynamically stable, and a new laparoscopy was performed. At this time, laparoscopy revealed hemoperitoneum of approximately 900mL. There was an ectopic pregnancy with active bleeding on the right tube and salpingectomy was performed (Figure 2). The postoperative follow-up was uneventful, and the patient was discharged on the same day. The pathology report confirmed the diagnosis of bilateral ectopic pregnancies, showing blood clot admixed with chorionic villi in the tissue removed from the left ovary and right tube. After having passed through the disorder of the event, patient chose not to perform IVF again. Nowadays patient opted for adoption and is currently satisfied with her decision.

## DISCUSSION

This study presented the case of a patient with bilateral ectopic pregnancy with a one-week interval between the diagnoses after IVF. It is known that the prevalence of ectopic pregnancy following IVF ranges between 2.1% and 9.4% of all clinical pregnancies (Azem *et al.* 1993; Edelstein & Morgan, 1989), with an increase in incidence probably related to high prevalence of tubal disease, previous abdominal surgery and pelvic inflammatory disease. Studies have found that ART procedures are also independently related to ectopic pregnancy (Clayton *et al*., 2006). The incidence of tubal disease in the donor egg recipient population is thought to be significantly lower than in the standard IVF population. Clayton *et al*. (2006) reviewed population-based data of pregnancies conceived with ART in United States clinics between 1999 and 2001. They demonstrated a significantly lower ectopic pregnancy rate in the fresh donor egg recipient population compared with the fresh non-donor IVF population (1.4% *vs.* 2.2%; odds ratio 0.63, 95% confidence interval 0.54-0.75).

Some studies have shown that ovulation induction, especially with clomiphene citrate, was an independent risk factor of ectopic pregnancy (Cohen *et al*., 1986; Marchbanks *et al*., 1985; Verhulst *et al*., 1993) and IVF did not increase further risk (Fernandez *et al.*, 1991). The contributing risk factors for the occurrence of ectopic pregnancy following IVF includes multiple embryo transfer and loading embryo(s) in a high volume of culture medium (Klipstein & Oskowitz, 2000; Nama & Manyonda, 2009). Bilateral ectopic pregnancy is the rarest kind of ectopic pregnancy with an estimated prevalence of 1/200000 spontaneous pregnancies (Polat *et al*., 2014). Also, multiple ovulations after induction could lead to multiple pregnancies, including bilateral ectopic pregnancies (De Los Rios *et al*., 2007). Since most of the cases of bilateral ectopic pregnancy are identified by laparoscopy (Zhu *et al*., 2014), the inspection of both fallopian tubes should not be forgotten during surgery (Ayoubi & Fanchin, 2003).

In our patient, careful inspection of both fallopian tubes was performed, and no signs of a developing ectopic pregnancy in the contralateral fallopian tube was seen, probably because it was still too underdeveloped to be diagnosed by laparoscopy at that moment. Mock *et al*. (2001) proposed to perform a systematic second sonography within one week when an ectopic pregnancy is medically or surgically treated. This protocol aimed to diagnose heterotopic pregnancy and reduce the need for emergency reintervention. Such protocol could also be applied for evaluation of contralateral adnexal masses. When assessing treatment options, the physician's experience, the clinical presentation and fertility expectation should be considered (Polat *et al*., 2014).

Medical treatment with methotrexate (a folic acid antagonist highly toxic to rapidly replicating tissues) can be attempted when the patient is hemodynamically stable with no evidence of acute intraperitoneal bleeding, serum β-HCG level less than 5000mIU/ml, absence of fetal cardiac activity and ectopic mass measuring less than 4cm in diameter (Practice Committee of American Society for Reproductive Medicine, 2008; Lipscomb *et al*., 1998). Treatment options must be fully explained and detailed before treatment decision. In conclusion, we have reported a case of bilateral ectopic pregnancy presented within one-week apart from the first surgery. The clinician should be aware that the treatment of one ectopic pregnancy does not preclude the occurrence of a second ectopic pregnancy in the same patient and should pay attention to the intra-operatory inspection of both side fallopian tubes in any ectopic pregnancy case (Zhu *et al*., 2014). Routine ultrasound after ectopic pregnancy treatment may be reasonable, especially in high risk patients.
